# Trends in colon and rectal cancer mortality in Australia from 1972 to 2015 and associated projections to 2040

**DOI:** 10.1038/s41598-022-07797-x

**Published:** 2022-03-07

**Authors:** Qingwei Luo, Jie-Bin Lew, Julia Steinberg, Joachim Worthington, Xue Qin Yu, Michael Caruana, Isabelle Soerjomataram, Freddie Bray, Sheena Lawrance, Maria Arcorace, Dianne L. O’Connell, Karen Canfell, Eleonora Feletto

**Affiliations:** 1 The Daffodil Centre, The University of Sydney, A Joint Venture with Cancer Council NSW, NSW 1340 Sydney, Australia; 2 Cancer Surveillance Branch, International Agency for Research on Cancer, Lyon, France; 3 New South Wales Cancer Registry, Cancer Institute NSW, Sydney, NSW Australia; 4 School of Medicine and Public Health, University of Newcastle, Newcastle, NSW Australia

**Keywords:** Cancer epidemiology, Cancer screening, Gastrointestinal cancer

## Abstract

Previously published sub-site Australian projections for colon and rectal cancers to 2035 using the World Health Organization’s mortality database sourced from the Australian Bureau of Statistics (ABS) predicted mortality rate decreases for colon cancer and increases for rectal cancer. There are complexities related to the interpretation of ABS’s Australian colon and rectal cancer mortality rates, which could lead to possible inaccuracies in mortality rates for these sub-sites. The largest Australian population-wide registry, New South Wales Cancer Registry (NSWCR), compares routinely-reported causes of death with the recorded medical history from multiple data sources. Therefore, this study used the NSWCR data to project mortality rates for colon and rectal cancers separately to 2040 in Australia. The mortality rates for colon cancer are projected to continuously decline over the period 2015–2040, from 7.0 to 4.7 per 100,000 males, and from 5.3 to 3.2 per 100,000 females. Similar decreasing trends in mortality rates for rectal cancer were projected over the period 2015–2040, from 4.9 to 3.7 per 100,000 males, and from 2.6 to 2.3 per 100,000 females. These projections provide benchmark estimates for the colorectal cancer burden in Australia against which the effectiveness of cancer control interventions can be measured.

## Introduction

Colorectal cancer, also known as bowel cancer, was the second most common cause of cancer death in Australia in 2016. Colorectal cancer mortality trends have decreased since the 1990s, and 5-year relative survival increased from 51.7% in 1986–1990 to 69.9% in 2011–2015^1^. The only national cancer mortality data available are recorded by the Australian Bureau of Statistics (ABS)^1^. Using these ABS colon and rectal cancer sub-site mortality data, a global study reported mortality trends with a significant decrease for colon cancer and significant increases for rectal cancer from the mid 2000s to 2013, with a projected 59% increase in the rectal cancer mortality rate from 2013 to 2035^2^. The complexities in recording cause of death and the corresponding interpretation of colorectal cancer mortality trends have been previously highlighted^3^. As 2.3–23.0% of colorectal cancer deaths each year are reported on the death certificate as ’bowel cancer’^1,3^, these deaths cannot be attributed specifically to colon or rectal cancer when coded by the ABS. Therefore, patterns in mortality by sub-site using ABS cancer mortality data have been based on incomplete reporting and may not reflect the true mortality patterns. To our knowledge, no studies have examined these data quality issues and their impact on temporal trends for each sub-site.

Data for all cancer patients in New South Wales (NSW) are available from the population-wide NSW Cancer Registry (NSWCR). The NSWCR collects and validates cancer registrations using a complete patient history based on multiple sources including public and private hospitals, radiation oncology departments, cancer care centres, pathology laboratories and other health services^4^. As multiple data sources are used, the NSWCR cancer mortality data can provide more reliable information on the trends in mortality rates for colon and rectal cancers separately^4–6^. The NSWCR is the largest state-based population-wide registry in Australia and its data may be considered nationally representative, as NSW is the most populous state in Australia with almost one third of the total population (8.2 million in 2020)^7^. Additionally, the NSW colorectal cancer mortality rates are almost identical to the national rates^1^. Hence, due to more complete reporting, the NSWCR mortality data for each of colon and rectal cancers allows for an alternative evaluation of trends in the Australian mortality rates for colon and rectal cancers.

In this study we used the state-level (NSWCR) sub-site data to examine temporal trends and to provide updated projections of mortality rates for colon and rectal cancers to 2040 in Australia. In addition, we investigated the potential effects of how deaths from colon and rectal cancers are coded in the ABS mortality data, by comparing data for colon and rectal cancer mortality from the ABS and NSWCR, and we evaluated the completeness of the national sub-site mortality data.

## Methods

### Data sources

#### Cancer mortality data from the ABS

National tabulated data on the numbers of colorectal cancer deaths using International Classification of Diseases (ICD-10) codes^8^ (ICD-10 codes C18-C20 and C26.0 for colorectal cancer, C18 for colon cancer, and C19-20 for rectal cancer) by sex, 5-year age group and calendar year for 1972–2015 were obtained from the Australian Institute of Health and Welfare (AIHW)^1^. Deaths for anal cancer (C21) were not included in this study for consistency with the standard reporting of Australian cancer statistics^1^. In Australia, cancer is a reportable cause of death with data released by the ABS sourced from the individual State- and Territory-administered Registries of Births, Deaths and Marriages (RBDMs)^1^. The ABS processes, codes and validates the information on causes of death from the death notifications provided by either the medical practitioner certifying the death or the coroner if there is a coronial investigation. Death registration is the only source of information on the cause of death used by the ABS (Appendix 1)^5^. In practice, a colorectal cancer death may be coded as C26.0 (malignant neoplasm of the intestinal tract unspecified) if recorded as ‘bowel cancer’. In 2019, the ABS retrospectively updated mortality data to include C26.0 in the colorectal cancer mortality data from 1968 to 2016 (accounting for 9.9%, range 2.3–23.0%, of the total number of colorectal cancer deaths). However, these deaths are not able to be separated for inclusion in the ABS colon and rectal cancer mortality data^3^.

#### Cancer mortality data from the NSWCR

The NSW sex-age-specific mortality rates for colorectal cancer (ICD-10 codes C18–C20 for colorectal cancer, C18 for colon cancer, and C19–20 for rectal cancer) were obtained from the NSWCR for 1972–2015 (most recent data available for this study). As multiple data sources are used, the cause of death information from the RBDM and the ABS can be compared to the patient’s medical history received by the NSWCR, allowing for more accurate coding of cause of death (Appendix 1)^4–6^. Due to the data verification processes undertaken by the NSWCR only a very small number of deaths are coded using the unspecified code C26.0 (e.g. 27 for C26.0 compared with 1,059 colon cancer deaths (C18) and 637 rectal cancer deaths (C19–20) in 2015), so this code was not included^9^.

#### Additional data

To facilitate the evaluation of the quality of the ABS mortality data, we obtained the number of new colorectal cancer cases and 5-year relative survival estimates by sex from 1993 to 2017 from the AIHW^1^. Population data for Australia and NSW by sex, 5-year age group and calendar year for 1972–2040 were obtained from the Australian Population Projections (Series B, based on medium population growth) produced by the ABS^10,11^.

### Statistical analyses

#### Assessment of quality of ABS mortality data for colon and rectal cancers

We first examined the observed age-standardised mortality rates for colorectal cancer, and for colon and rectal cancers separately, using data from the ABS and NSWCR (Appendix 2 Fig. S2.1). The data were then evaluated using the mortality-to-incidence (M:I) ratio method, which is considered to be a good indicator for assessing the completeness of cancer incidence data as an independent case ascertainment method^12^. The M:I ratio is a comparison of the number of deaths which were obtained from a source independent of the cancer registry with the number of incident cases from the cancer registry for the same period. As the accuracy of cancer incidence data in Australia is known to be high^13^, the M:I ratio may be used to evaluate the completeness of the recording of cancer specific causes of death by the ABS. As the M:I ratio and 1 minus the estimated 5-year relative survival (1-survival) are different measures, and both the incidence and survival for colorectal cancer changed over time, we focussed on consistent trends rather than exact agreement between the two measures. Therefore, we calculated the M:I ratio and compared it with (1-survival) for each 5-year period, and the Pearson correlation coefficient was calculated to measure the association between the M:I ratio and (1-survival)^14^. A positive correlation between the two measures indicates the completeness of data. For example, with a decreasing incidence and increasing survival, the M:I ratio is expected to decrease. For national data, we calculated the M:I ratio using the ABS mortality data and incidence data from the Australian cancer registries, which are independent of the ABS mortality data^1^.

#### Statistical projections

Colon and rectal cancer mortality projections for this study were based on the NSWCR data which were used as an alternative data source for estimating the national rates. The observed and projected numbers of deaths were estimated by applying the NSW sex-age-specific mortality rates to the whole Australian population. Age-period-cohort (APC) models are increasingly used to project cancer mortality rates^15^, as they capture the age, period and cohort effects on mortality, which reflect diagnostic and treatment factors that may impact survival and exposure to risk factors that may impact incidence and ultimately mortality^16^. In this analysis APC models were fitted by the apcspline command in Stata 16 with natural cubic splines for smoothing. Details of the methods used are provided elsewhere^15^ and are summarised in Appendix 3. The Australian National Bowel Cancer Screening Program (NBCSP) underwent phased implementation from 2006 to 2020^17^ and now targets Australians from age 50 and then biennially to age 74^18^. We therefore developed separate APC models for people aged less than 50 years and for those aged 50 years and older (Appendix 3). We also report the observed and projected numbers of deaths for three age categories, under 50, 50–74 and 75+, to highlight trends for those eligible for the NBCSP.

All statistical analyses were performed using Stata (version 16, Stata Corporation, College Station, TX). Mortality rates were age-standardised using the Segi World standard population for the main results to facilitate a comparison with the global study by Araghi et al.^2^ Age-standardised rates using the 2001 Australian population were estimated in a supplementary analysis.

### Ethics approval

This population-based study used existing tabulated data for colorectal cancer incidence, mortality and survival released by the Australian Institute of Health and Welfare and the New South Wales Cancer Registry. Ethics approval and patient consent was not required to use these aggregated data. All methods were carried out in accordance with relevant guidelines and regulations.

## Results

Over the period 1972–2015, a total of 191,968 colorectal cancer (colon 118,889; rectum 54,100; C26.0 deaths 18,979) deaths recorded in the ABS, and 63,675 colorectal cancer (41,889 colon; 21,786 rectum) deaths recorded in the NSWCR were included in this study.

### Evaluation of the quality of the ABS mortality data for colon and rectal cancers

**Figure 1:**
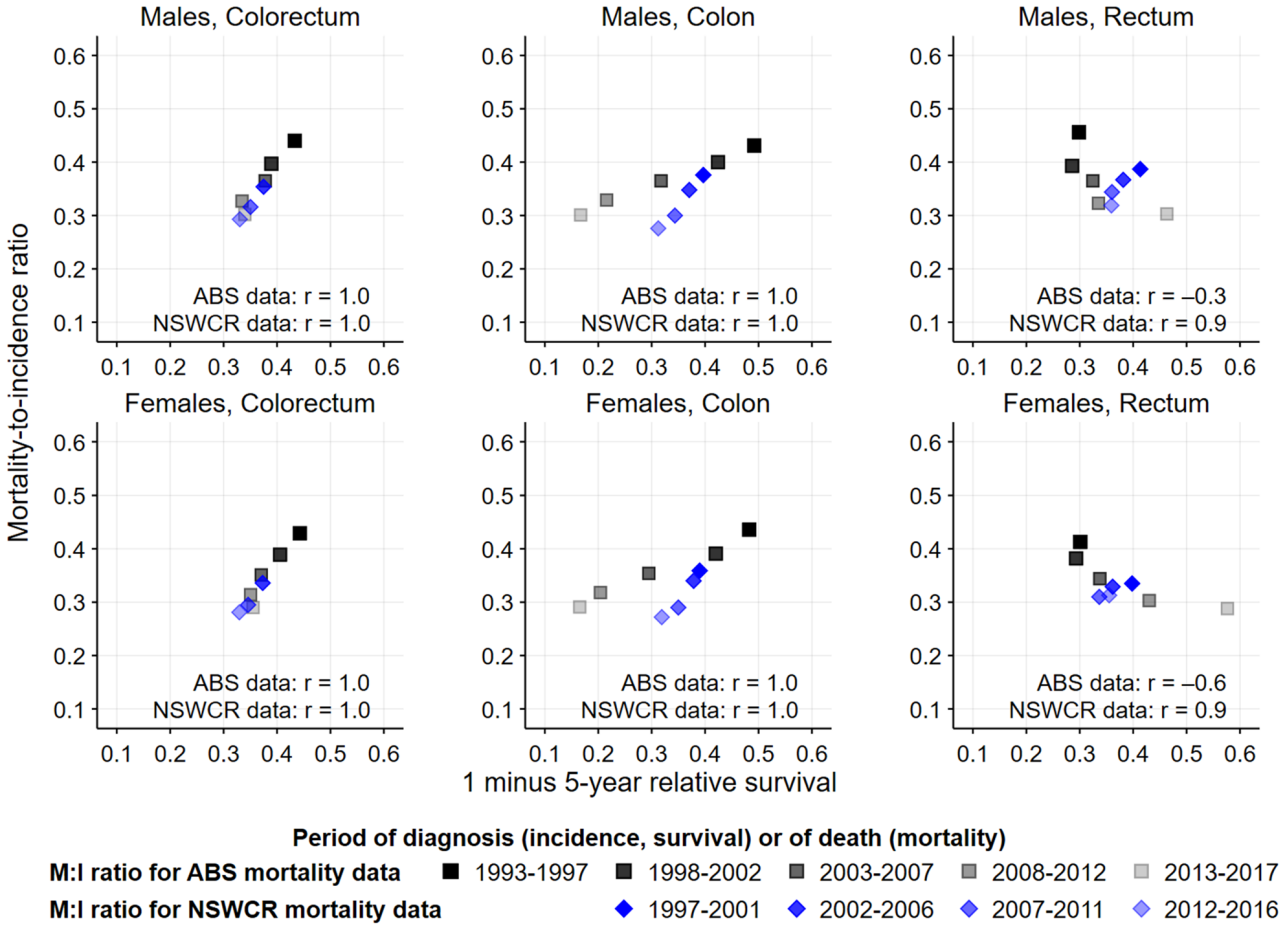
Comparison of mortality-to-incidence ratio (M:I ratio) and (1 minus 5-year relative survival) for colon and rectal cancers in Australia using mortality data from the Australian Bureau of Statistics (ABS) and the New South Wales Cancer Registry (NSWCR). r denotes the correlation coefficient. The M:I ratio for the ABS mortality data used incidence data from the Australian cancer registries (Reference: Australian Institute of Health and Welfare (AIHW)^1^). The M:I ratio for the NSWCR mortality data used the NSWCR incidence data. Some validation of the NSWCR mortality data is partially based on pathological reports and other medical records received by the NSWCR, so the NSWCR mortality data are not completely independent of the incidence data.

Figure 1 shows the association between the M:I ratio and (1-survival) for colorectal cancer combined (including C26.0), and for colon and rectal cancers separately, which do not include deaths coded as C26.0. For both males and females there is a strong positive correlation between the M:I ratio and (1-survival) for each of colorectal cancer combined and colon cancer using mortality data from the ABS (all correlation coefficients equal to 1.0), indicating the completeness of ABS mortality data for colorectal cancer combined and colon cancer. However, a negative correlation between the M:I ratio and (1-survival) was observed for rectal cancer (correlation coefficient: − 0.3 for males and − 0.6 for females) (Fig. 1). This indicates that the ABS rectal cancer mortality data are not accurate. There is a strong positive correlation between the M:I ratio and (1-survival) for each of colon and rectal cancers using the NSWCR mortality data (correlation coefficient: 1.0 for colorectal and colon cancer, 0.9 for rectal cancer) (Fig. 1). It is acknowledged that the NSWCR mortality data cannot be considered completely independent of the NSWCR incidence data. However, the consistency between the M:I ratio and (1-survival) provides insight into the reliability of the mortality data from the NSWCR.

### Trends and projections of colon and rectal cancer mortality rates

**Figure 2:**
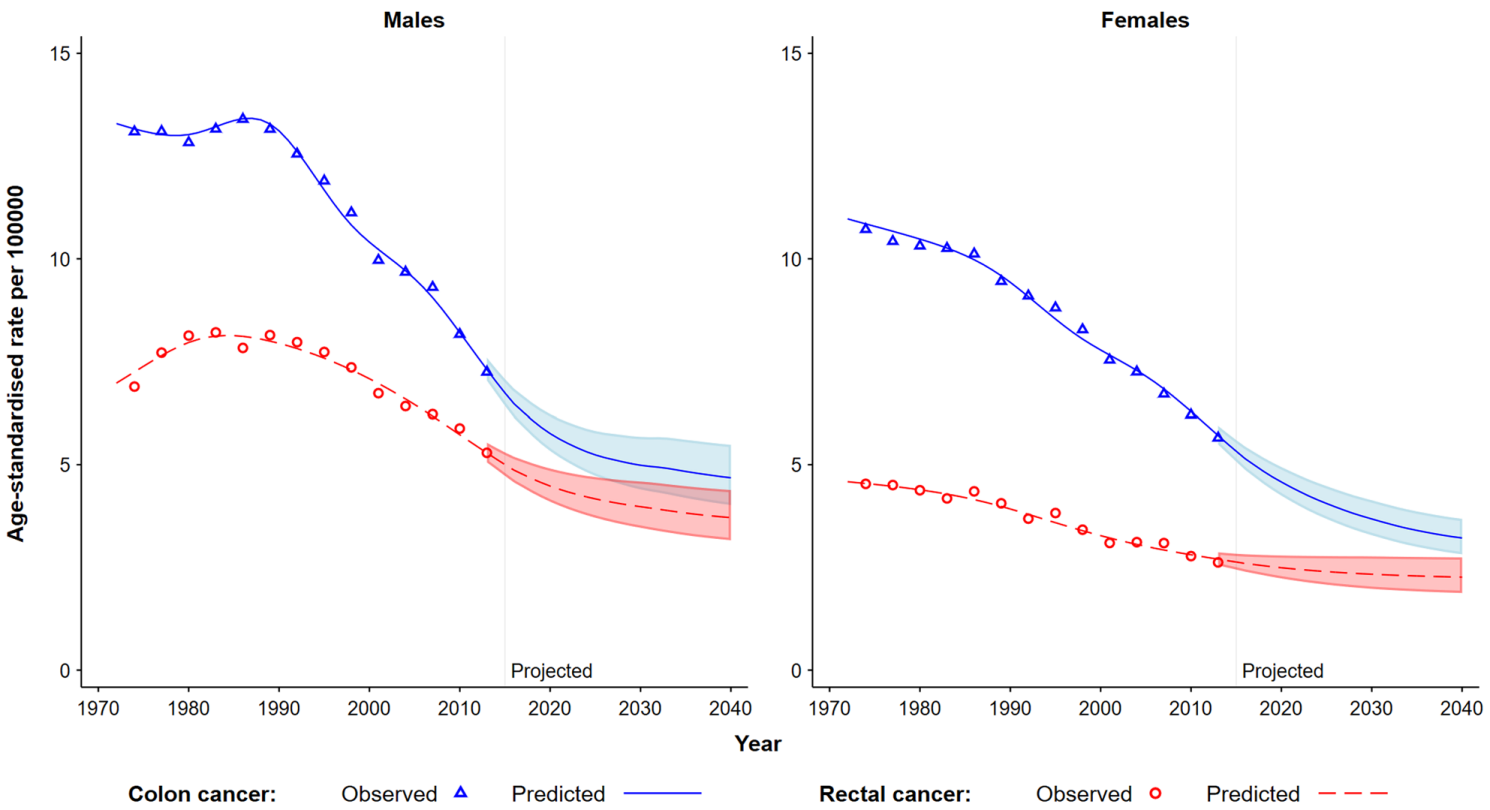
Observed and predicted age-standardised mortality rates for colon and rectal cancers by sex in Australia, 1972–2040. Observed and predicted mortality rates were based on the data from the New South Wales Cancer Registry. All rates are age-standardised using the Segi World standard population. The shaded area represents the 95% uncertainty interval.

The observed and predicted sex-specific age-standardised mortality rates for colon and rectal cancers based on the NSWCR data are presented in Fig. 2. For both colon and rectal cancers, different sex-specific patterns in mortality rates were observed, with the mortality rates being lower for females than for males. For females, the overall mortality rates for both colon and rectal cancers showed steady declines over the observed period from 1972 to 2015, while for males the declines occurred from the 1990s. These sex differentials in colon and rectal cancer mortality became smaller over time, but for both colon and rectal cancers the mortality rates for males are expected to be higher than those for females in 2040 (Fig. 2 and Table 1).

**Table 1:** Observed and projected age-standardised mortality rates for colon and rectal cancers to 2040 in Australia.

	Age-standardised mortality rates per 100,000 for colon and rectal cancers^a^										
	Observed			% change from 1972 to 2015	% change from 2005 to 2015	Projected					% change from 2015 to 2040
	1972	2005	2015			2020	2025	2030	2035	2040	
**Colon cancer**											
**Overall**	11.6	8.4	6.1	− 47.4	− 27.4	5.1 (4.8–5.6)	4.6 (4.2–5.1)	4.3 (3.8–4.9)	4.1 (3.6–4.7)	3.9 (3.4–4.6)	− 36.1
< 50 years	1.5	0.6	0.7	− 53.3	16.7	0.8 (0.7–0.9)	0.9 (0.8–1.2)	1.1 (0.9–1.4)	1.2 (1.0–1.6)	1.3 (1.0–1.6)	85.7
50–74 years	39.8	29.4	18.7	− 53.0	− 36.4	14.6 (13.7–15.6)	12.5 (11.4–13.6)	11.3 (10.3–12.5)	10.8 (9.7–11.9)	10.4 (9.3–11.6)	− 44.4
75+	162.0	132.2	109.7	− 32.3	− 17.0	94.6 (89.2–100.3)	81.4 (75.5–87.7)	69.2 (63.4–75.4)	59.0 (53.6–65.0)	52.4 (47.2–58.2)	− 52.2
**Males**	12.4	9.7	7.0	− 43.5	− 27.8	5.8 (5.3–6.2)	5.2 (4.7–5.8)	5.0 (4.4–5.7)	4.8 (4.2–5.6)	4.7 (4.0–5.5)	− 32.9
< 50 years	1.5	0.6	0.9	− 40.0	50.0	1.0 (0.8–1.1)	1.3 (1.0–1.5)	1.6 (1.2–2.0)	1.8 (1.4–2.3)	1.8 (1.4–2.4)	100.0
50–74 years	42.9	34.4	21.8	− 49.2	− 36.6	16.5 (15.5–17.6)	14.0 (12.8–15.2)	12.6 (11.4–13.8)	11.9 (10.7–13.2)	11.5 (10.3–12.8)	− 47.2
75+	172.6	154.5	119.3	− 30.9	− 22.8	100.7 (94.8–106.8)	86.2 (79.9–92.9)	73.9 (67.7–80.6)	63.8 (58.0–70.2)	57.2 (51.6–63.4)	− 52.1
**Females**	11.0	7.2	5.3	− 51.8	− 26.4	4.6 (4.2–4.9)	4.1 (3.7–4.5)	3.7 (3.3–4.1)	3.4 (3.0–3.9)	3.2 (2.8–3.7)	− 39.6
< 50 years	1.5	0.6	0.5	− 66.7	− 16.7	0.6 (0.5–0.7)	0.7 (0.5–0.8)	0.7 (0.5–0.9)	0.7 (0.5–0.9)	0.7 (0.5–0.9)	40.0
50–74 years	37.2	24.6	15.8	− 57.5	− 35.8	12.8 (12.0–13.7)	11.1 (10.1–12.1)	10.1 (9.2–11.2)	9.7 (8.7–10.8)	9.4 (8.5–10.6)	− 40.5
75+	155.3	116.9	101.9	− 34.4	− 12.8	89.5 (84.4–94.9)	77.2 (71.6–83.2)	65.2 (59.7–71.1)	55.1 (50.0–60.7)	48.5 (43.6–53.9)	− 52.4
**Rectal cancer**											
**Overall**	5.5	4.6	3.7	− 32.7	− 19.6	3.4 (3.1–3.8)	3.2 (2.9–3.7)	3.1 (2.7–3.6)	3.0 (2.6–3.6)	3.0 (2.5–3.5)	− 18.9
< 50 years	0.6	0.5	0.5	− 16.7	0.0	0.5 (0.4–0.7)	0.6 (0.5–0.8)	0.6 (0.5–0.9)	0.7 (0.5–0.9)	0.7 (0.5–0.9)	40.0
50–74 years	18.9	17.1	12.3	− 34.9	− 28.1	11.4 (10.5–12.4)	10.4 (9.4–11.6)	9.9 (8.7–11.2)	9.6 (8.4–11.0)	9.4 (8.2–10.9)	− 23.6
75+	79.6	58.5	52.2	− 34.4	− 10.8	47.8 (44.1–51.8)	44.3 (40.0–49.1)	41.1 (36.5–46.2)	38.3 (33.6–43.6)	36.3 (31.6–41.7)	− 30.5
**Males**	7.0	6.4	4.9	− 30.0	− 23.4	4.5 (4.1–4.9)	4.2 (3.7–4.7)	4.0 (3.5–4.6)	3.8 (3.3–4.5)	3.7 (3.2–4.4)	− 24.5
< 50 years	0.7	0.5	0.5	− 28.6	0.0	0.7 (0.6–0.8)	0.8 (0.6–1.0)	0.9 (0.7–1.2)	1.0 (0.7–1.3)	1.0 (0.7–1.3)	100.0
50–74 years	23.4	23.8	16.5	− 29.5	− 30.7	14.6 (13.6–15.8)	13.1 (11.9–14.4)	12.1 (10.9–13.6)	11.7 (10.4–13.2)	11.4 (10.1–12.9)	− 30.9
75+	108.0	86.5	72.7	− 32.7	− 16.0	65.5 (60.9–70.4)	58.9 (53.7–64.5)	52.9 (47.7–58.8)	47.8 (42.6–53.6)	44.2 (39.1–49.9)	− 39.2
**Females**	4.4	3.1	2.6	− 40.9	− 16.1	2.5 (2.2–2.8)	2.4 (2.1–2.8)	2.3 (2.0–2.8)	2.3 (1.9–2.8)	2.3 (1.9–2.7)	− 11.5
< 50 years	0.5	0.4	0.5	0.0	25.0	0.4 (0.3–0.5)	0.4 (0.3–0.5)	0.4 (0.3–0.5)	0.4 (0.3–0.5)	0.4 (0.3–0.5)	− 20.0
50–74 years	15.1	10.6	8.2	− 45.7	− 22.6	8.3 (7.6–9.2)	8.0 (7.1–9.1)	7.8 (6.8–9.0)	7.7 (6.6–9.0)	7.6 (6.5–8.9)	− 7.3
75+	64.8	39.4	36.9	− 43.1	− 6.3	33.9 (30.9–37.2)	32.5 (28.9–36.6)	31.4 (27.4–36.0)	30.6 (26.3–35.5)	30.0 (25.6–35.1)	− 18.7

^a^Observed and projected mortality rates were based on the data from the New South Wales Cancer Registry. All rates are age-standardised using the Segi standard population.

The overall age-standardised mortality rates for both colon and rectal cancers decreased by 47.4% and 32.7% over the observed period from 1972 to 2015, respectively. The decreases were more pronounced from 2005 to 2015 with the age-standardised mortality rates decreased by 27.4% for colon cancer and by 19.6% for rectal cancer (Table 1). The age-standardised mortality rates for colon cancer are projected to continuously decline over the period 2015–2040, from 7.0 to 4.7 per 100,000 males (by 32.9%), and from 5.3 to 3.2 per 100,000 females (by 39.6%) (Fig. 2 and Table 1). Similar decreasing trends in age-standardised mortality rates for rectal cancer were projected over the period 2015–2040, from 4.9 to 3.7 per 100,000 males (by 24.5%), and from 2.6 to 2.3 per 100,000 females (by 11.5%) (Fig. 2 and Table 1).

**Figure 3:**
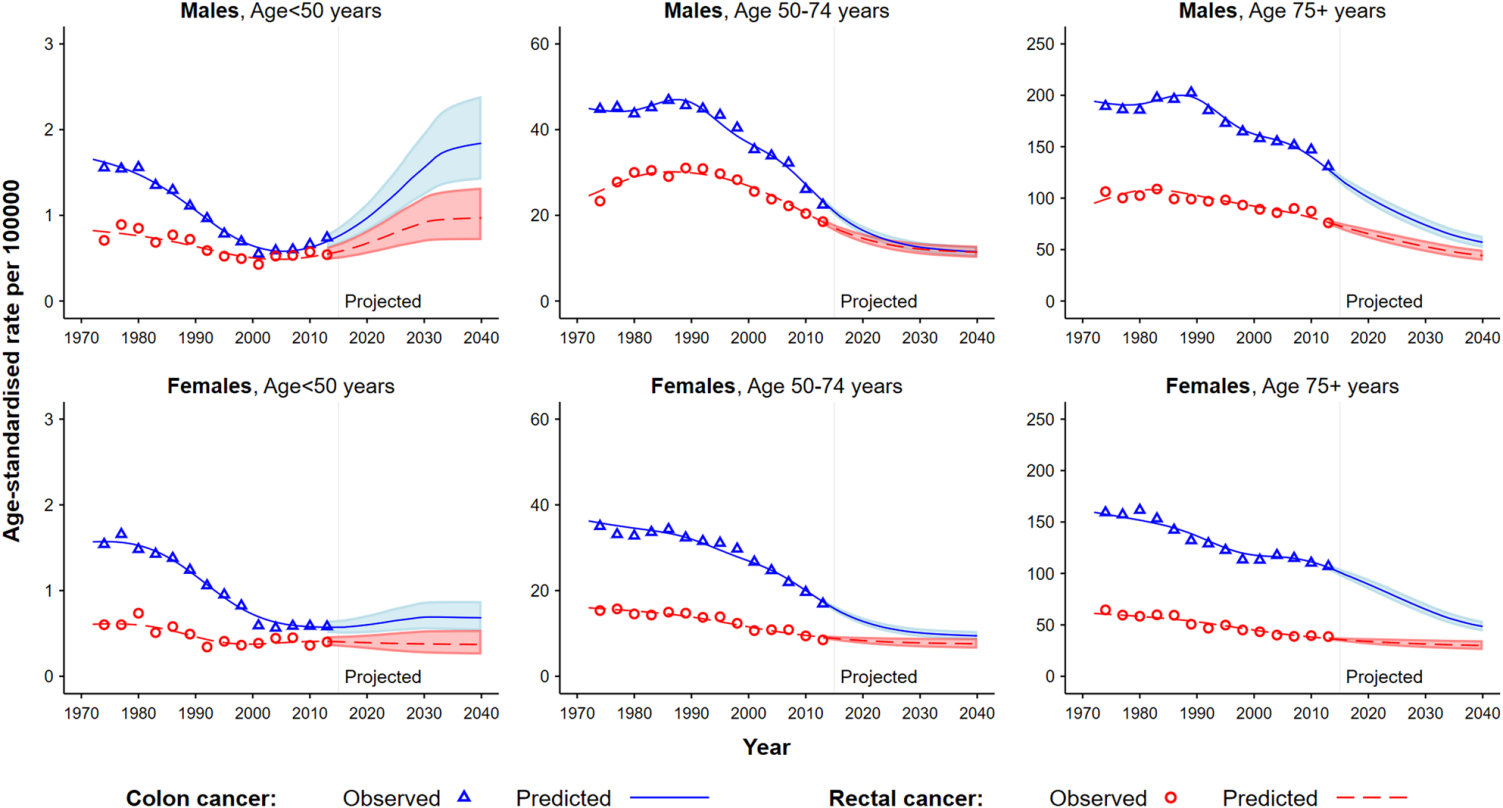
Observed and predicted age-standardised mortality rates for colon and rectal cancers by sex and age group in Australia, 1972–2040. Observed and predicted mortality rates were based on the data from the New South Wales Cancer Registry. All rates are age-standardised using the Segi World standard population. The shaded area represents the 95% uncertainty interval.

**Figure 4:**
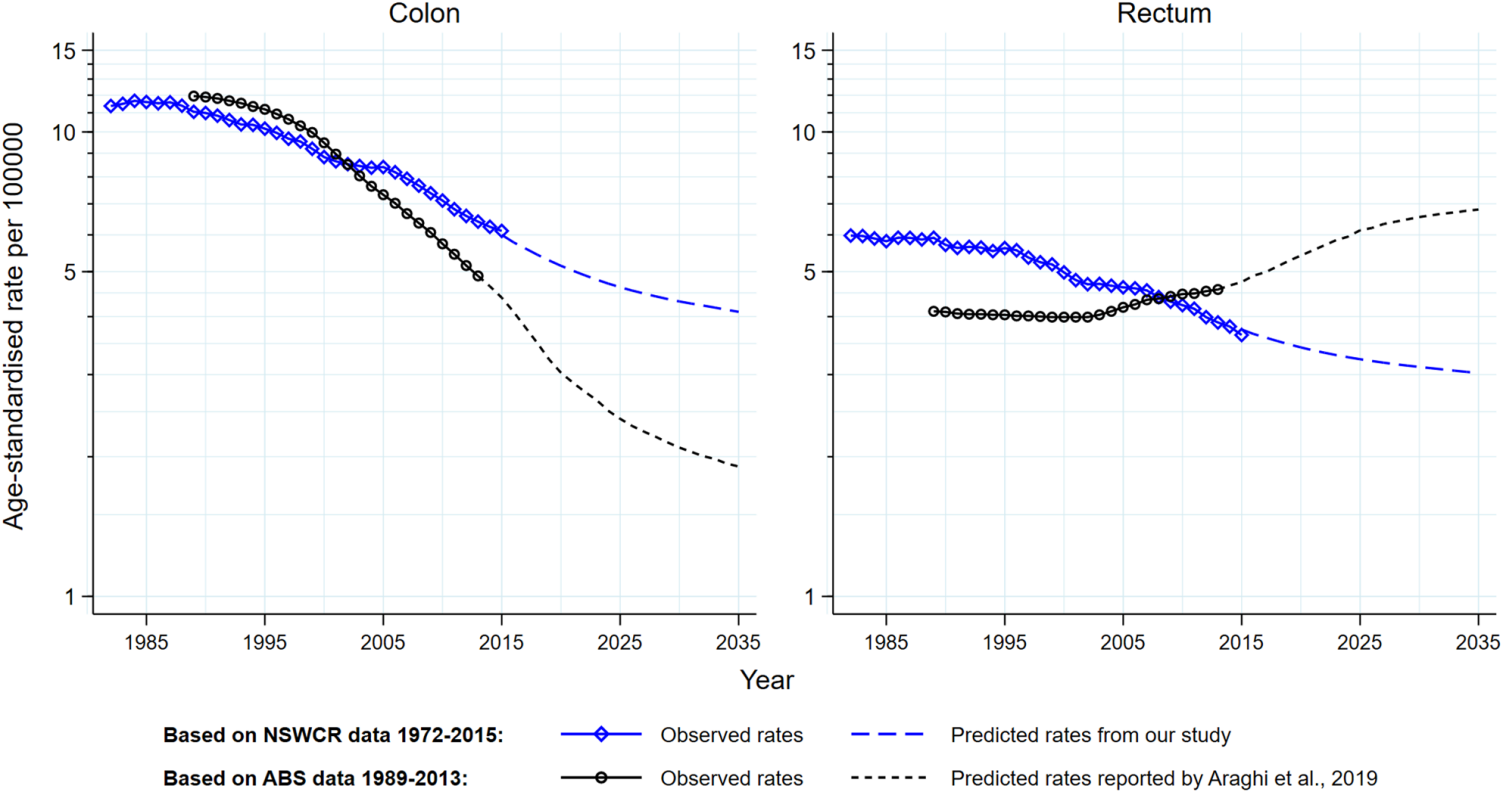
Comparison of projected age-standardised mortality rates published by Araghi et al.^2^, with the results from this study and observed rates based on data from the NSWCR for colon and rectal cancers, males and females combined. All rates are age-standardised using the Segi World standard population. NSWCR: New South Wales Cancer Registry. ABS: Australian Bureau of Statistics. Age-standardised rates from Araghi et al.^2^ were extracted from the published table and figure (using online computer software WebPlotDigitizer which is available via https://automeris.io/WebPlotDigitizer/). Reference: Araghi et al.^2^.

When looking at age-stratified standardised rates for colon and rectal cancers (Fig. 3 and Table 1), for both males and females the rates were low for those aged less than 50 years (less than 3 per 100,000), with an increasing trend for males from the year 2000 and projected to 2040, but rates levelling off for females from the early 2000s. The mortality rates for those aged 50 years and over showed a steady decline from the 1990s across both sub-sites and sexes, and are expected to continue decreasing to 2040, albeit with the decline occurring at a slower pace for females (Fig. 3 and Table 1). Age-standardised mortality rates standardised using the 2001 Australian population show a similar pattern (Appendix 4). Comparisons with previously published colon and rectal cancer projections for Australia are reported in Fig. 4.

The observed and projected numbers of deaths estimated by applying the NSW sex and age-specific mortality rates to the whole Australian population are presented in Table 2. Over the period 2015–2040, the numbers of deaths from colon cancer are expected to be relatively stable, with the total number of deaths increasing slightly from 3193 (1614 males and 1579 females) in 2015 to 3269 (1711 males and 1558 females) in 2040 (Table 2). The pattern for colon cancer mortality differs markedly by age group for both males and females. There was an increase in the number of deaths for those under 50, but a significant reduction in the number of deaths from colon cancer for those 50–74 years. For rectal cancer, the number of deaths is projected to increase from 1751 (1086 males and 665 females) in 2015 to 2370 (1353 males and 1017 females) in 2040 (Table 2). The pattern for rectal cancer deaths also differs by sex and age group. For males, the number of rectal cancer deaths increased for those under 50 and for the 75 and over age group, but are expected to decrease slightly for those aged 50–74 years. For females, the number of rectal cancer deaths increased for all three age categories (Table 2).

**Table 2:** Observed and projected numbers of deaths from colon and rectal cancers to 2040 in Australia.

	Number of deaths from colon and rectal cancers^a^						% change from 2015 to 2040	Total in 2016–2040
	Observed	Projected						
	2015	2020	2025	2030	2035	2040		
**Colon cancer**								
Overall								
All ages	3193	3096 (2894–3314)	3120 (2860–3408)	3190 (2879–3541)	3248 (2898–3648)	3269 (2893–3703)	2.4	79,374 (72,338–87,270)
< 50 years	146	177 (149–210)	227 (185–279)	297 (235–374)	348 (271–447)	376 (290–487)	157.5	6652 (5318–8333)
50–74 years	1251	1124 (1053–1201)	1020 (938–1110)	975 (885–1075)	969 (873–1076)	977 (876–1090)	− 21.9	25,783 (23,667–28,100)
75+	1796	1795 (1692–1904)	1873 (1737–2019)	1918 (1759–2092)	1931 (1754–2125)	1916 (1727–2125)	6.7	46,939 (43,353–50,838)
Males								
All ages	1614	1551 (1448–1662)	1580 (1444–1730)	1639 (1474–1828)	1688 (1498–1908)	1711 (1505–1951)	6.0	40,585 (36,851–44,813)
< 50 years	88	108 (92–128)	149 (121–182)	204 (162–257)	249 (193–320)	273 (210–355)	210.3	4530 (3613–5689)
50–74 years	715	621 (582–662)	555 (511–603)	526 (478–579)	519 (468–575)	520 (467–579)	− 27.3	14,017 (12,888–15,251)
75+	811	821 (774–872)	876 (812–944)	909 (834–991)	921 (837–1013)	918 (828–1017)	13.2	22,038 (20,350–23,873)
Females								
All ages	1579	1545 (1446–1652)	1541 (1415–1678)	1551 (1405–1713)	1559 (1399–1740)	1558 (1388–1752)	− 1.3	38,789 (35,487–42,458)
< 50 years	58	68 (57–81)	78 (64–97)	93 (73–117)	99 (78–127)	103 (80–132)	77.3	2122 (1705–2644)
50–74 years	536	503 (471–538)	465 (427–507)	449 (407–496)	450 (405–501)	457 (409–511)	− 14.7	11,766 (10,779–12,849)
75+	985	973 (918–1032)	997 (925–1074)	1009 (925–1101)	1010 (917–1112)	998 (899–1109)	1.3	24,901 (23,003–26,965)
**Rectal cancer**								
Overall								
All ages	1751	1872 (1712–2050)	1992 (1776–2238)	2134 (1867–2445)	2262 (1956–2625)	2370 (2032–2773)	35.3	52,004 (46,031–58,933)
< 50 years	106	121 (98–149)	141 (109–184)	171 (127–232)	189 (137–261)	201 (145–280)	89.6	3920 (2963–5207)
50–74 years	807	859 (791–934)	841 (756–936)	841 (744–952)	853 (746–975)	873 (759–1005)	8.2	21,295 (19,068–23,803)
75+	838	892 (823–967)	1009 (911–1118)	1121 (997–1261)	1220 (1072–1389)	1296 (1128–1488)	54.6	26,788 (23,999–29,923)
Males								
All ages	1086	1150 (1060–1249)	1205 (1085–1340)	1268 (1122–1437)	1317 (1152–1510)	1353 (1174–1564)	24.6	31,003 (27,740–34,751)
< 50 years	57	75 (61–92)	95 (74–121)	120 (90–159)	135 (100–182)	144 (106–197)	153.5	2675 (2047–3504)
50–74 years	536	542 (503–584)	512 (465–563)	501 (449–559)	502 (446–566)	510 (450–578)	− 4.8	12,923 (11,717–14,261)
75+	493	533 (495–573)	599 (546–656)	647 (583–719)	680 (606–762)	698 (618–789)	41.6	15,405 (13,976–16,986)
Females								
All ages	665	723 (652–801)	787 (691–898)	865 (745–1007)	946 (804–1115)	1017 (858–1209)	52.9	21,001 (18,291–24,182)
< 50 years	49	45 (37–56)	47 (35–63)	51 (36–73)	54 (38–79)	56 (39–83)	15.3	1245 (917–1703)
50–74 years	271	318 (288–350)	329 (291–373)	340 (295–392)	351 (300–409)	363 (309–427)	34.0	8372 (7351–9542)
75+	345	360 (328–394)	411 (365–462)	474 (414–542)	540 (466–627)	597 (510–700)	73.2	11,384 (10,023–12,937)

^a^The numbers of deaths were estimated by applying the NSW sex and age-specific mortality rates to the whole Australian population.

## Discussion

To our knowledge this is the first study to evaluate the complexities related to Australian colon and rectal cancer mortality data, and to derive long term projections using alternative state-level sub-site data. In this study, using the NSWCR data, our projections suggest a continuing decline in mortality rates in Australia over the period 2015–2040 for both colon and rectal cancers. Our study found consistent trends in mortality rates for colorectal cancer combined using data from the ABS or NSWCR, which confirmed the importance of including deaths coded as C26.0 when examining mortality rates for colorectal cancer using the ABS mortality data^3^. This supports the suggestion from the ABS that C26.0 deaths were predominantly colorectal cancer deaths^3^, although it is of course still not possible to examine accurate trends separately for the colon and rectal cancer sub-sites using the ABS mortality data.

The 2019 study by Araghi et al. reported global trends in colorectal cancer mortality and long-term projections to 2035 including for Australia^2^. The decrease in Australian rectal cancer mortality rates in the present study was in contrast to the increasing rectal cancer rates from the early 2000s to 2035 reported in the global study. The global study reported a 50% decrease in the colon cancer mortality rates and a 59% increase in the rectal cancer mortality rates from 2013 to 2035^2^. In the present study, a slower pace was projected for the decrease in colon cancer mortality rates (36%) and a 23% decrease in rectal cancer mortality rates over the same period (Appendix 5). While different methods were used for the projections, it is likely that these differences are primarily due to the different observed data on which the projections were based, with the previous study having relied on the incomplete ABS mortality data^2^. In addition, the mortality rate for rectal cancer as reported by Araghi et al. included anal cancer (C21)^2^, but this was not included in our study. Given the reported anal cancer mortality rates in Australia have been consistently low over the last two decades (age-standardised rate ≤ 0.2 deaths per 100,000), the inclusion of anal cancer in the global study would not materially contribute to the contrasting results for rectal cancer mortality^1^.

The overall decline in colon and rectal cancer mortality rates observed in this study were consistent with the observed overall decreasing trends in the incidence rates and increase in survival for both colon and rectal cancers in Australia (Appendix 6). The corresponding declining mortality trends for both colon and rectal cancers reported in this study for Australia are likely due to multiple factors, including the changing prevalence of risk factors, improvements in cancer treatment and increasing survival, and the advent of opportunistic colorectal cancer screening since the 1990s and a phased rollout of the NBCSP from 2006^19^. Notably, colorectal cancer screening is also a means of primary prevention through the identification and removal of pre-cancerous conditions^20^, and can reduce both colorectal cancer incidence and mortality in the population^20,21^. Our projections showed that in those aged over 50 the trends for both colon and rectal cancers were decreasing to 2040. Specifically, in the target group for the NBCSP, those aged 50–74 showed a projected decrease in age-standardised mortality rates for males and females. These projections could be considered conservative given the full effect of the NBCSP is not yet reflected in the observed data. Advances in management and treatment regimens for colorectal cancer in Australia may have also contributed to improved cancer survival and decreasing colorectal cancer mortality^22,23^. The 2005 clinical practice guidelines were updated in 2017 and more recent recommendations include adjuvant therapy for advanced stages of colon cancer and improved surgical treatment combined with neoadjuvant chemoradiation therapy or radiation therapy alone for advanced rectal cancer. Targeted treatments are also recommended for metastatic disease^22,23^. As this study examined mortality trends and projections, incidence projections and the impact of colorectal cancer treatment and management were not assessed in detail and warrant future research.

A previous study showed that over the last two decades colon and rectal cancer incidence rates increased for people aged less than 50 years in Australia^19^. Our projections also suggest that increasing colon and rectal cancer mortality rates for males under 50 years of age from the year 2000 will continue to 2040, and for females start to level off from the early 2000s. It is possible that changing trends in risk factor exposure could be the key drivers behind the increasing rates^23^. The prevalence of obesity and overweight has increased in Australia with over a quarter of children and adolescents overweight and obese in 2014–2015^24^. The data from the National Health Survey suggested that almost half of young Australians aged 18–24 were not sufficiently active (defined as more than 150 min of physical activity over 5 or more sessions in the previous week) in 2014–2015^25^. Other colorectal cancer risk factors are likely to have contributed, including alcohol consumption, and diet low in fibre and high in red or processed meats^19^, so additional research is required to determine the causal factors. This result also highlights the ongoing need to further evaluate the start age for the organised screening program, as it may be useful as an intervention to mediate the increasing mortality for people under 50 years of age^19^.

Our findings also supported the ABS’s caution that accurately quantifying the mortality rate for rectal cancer using ABS mortality data is complex^3^. This is illustrated through the marked differences observed between the trends in the rectal cancer mortality rates using the two data sources. There are two possible reasons for these differences. Firstly, C26.0 deaths recorded by the ABS cannot be classified by sub-site, which results in underestimates of deaths for both colon and rectal cancers. Secondly, a potential misclassification of cause of death for colon and rectal cancers in the ABS has been posited^26–28^. This potential misclassification is possibly due to changes in the ICD codes over time, as in the earlier ICD-9 code rectal cancer was defined as code 154 “Malignant neoplasm of rectum, rectosigmoid junction and anus”, which since 1999 has been separated into the ICD-10 codes C19 “Malignant neoplasm of the rectosigmoid junction”, C20 “Malignant neoplasm of the rectum” and C21 “Malignant neoplasm of anus and anal canal”. The rectosigmoid junction lies between the sigmoid colon and the rectum, so it is possible that some deaths coded as C19 are, in fact, colon cancer deaths^27^. This possible misclassification could lead to an underestimate of deaths from colon cancer and an overestimate of rectal cancer deaths. As data for C19 and C20 were not available separately in the ABS mortality data we were not able to determine the extent of any misclassification. For these reasons, analyses using cancer mortality data from the ABS to report trends for colon and rectal cancers separately should be interpreted with caution, and further education for medical practitioners who certify cause of death is recommended. Also, further investigation into the extent of the misclassification or miscoding of colorectal cancer data by sub-site in the ABS is warranted.

This study has limitations which should be considered when interpreting the results. Firstly, projections of colon and rectal cancer mortality rates reported in this study were based on the NSWCR data as a proxy for the national rates. However, NSW accounts for one third of the total Australian population with similar population structure^7^, and the NSW cancer incidence and mortality rates for most cancer types (including colorectal cancer) are generally consistent with national rates (Appendix 7)^1^. The NSW data can provide some insight into future Australian trends in mortality rates for each of colon and rectal cancers in the absence of reliable national data. Secondly, as with all modelled projections, the projections are dependent on the assumptions which imply a continuous effect over time. We assumed that the age effect remained unchanged over time and reflects the general level of cancer risk in the population, and that the future cohort and future period effects would have the same effect as those for the most recent observed cohort and period^15^. These assumptions do not aim to capture any major quantitative changes in any risk factors, especially for younger age groups. Inherent in the projections is the impact of opportunistic screening and current and emerging treatment regimens. In practice, the effect of such factors are likely to change over time, with the positive effects of current treatments and opportunistic screening perhaps being reduced over time or impacting only a small number of cases, while the introduction of any new treatments might improve survival outcomes and thus further reduce mortality rates. Additionally, it was not possible to incorporate detailed data on screening participation into our projection models, due to the lack of comprehensive national data available on opportunistic colorectal cancer screening, both before and after the implementation of the population-wide NBCSP, and because of the short period for which NBCSP screening data were available. Therefore, our projections are not able to reflect future screening practices. Detailed projections incorporating the impact of the NBCSP using microsimulation modelling have been published previously^21^. Future work combining our projections and microsimulation modelling would improve our projections of the colorectal cancer burden in Australia and allow for updated evaluations of colorectal cancer control initiatives. Furthermore, our projections do not capture the impact of the recent COVID-19 pandemic, which led to disruptions in healthcare provision that may contribute to future excess deaths^29,30^. Estimating these impacts will be the subject of our future work.

Despite these limitations, this study also has many strengths. Firstly, the long-term observed data from the NSWCR are known to be of high quality with excellent population coverage, and coded cause of death being verified using further information on patients’ medical history^4,6^. Secondly, this study used stratified APC models to project mortality rates, and provided separate estimations of age, period and cohort effects for people aged less than 50 years and those aged 50 years and older. These effects are used as surrogates for a range of factors that contribute to mortality, including changes in exposure to risk factors, cancer diagnosis methods, and screening and cancer management^16^. As opposing trends in colon and rectal cancer mortality have been reported for several countries^2^, with a misclassification of cause of death being a possible contributing factor, the method used in our study to validate the cause of death data could be applied to other population-based data where these trends are seen and high-quality data for incidence and survival are available.

This study provides more reliable projections for colon and rectal cancer mortality in Australia to 2040 than were previously available. In contrast to previous studies, our projections suggested that mortality rates for both colon and rectal cancers will continue to decrease in Australia, although the total numbers of colorectal cancer deaths will continue to increase due to the ageing population. These projections can help inform health service planning to meet the requirements for future cancer care and treatment in Australia, which can differ by sub-site. The results can also serve as a benchmark against which to measure the impact and effectiveness of colorectal cancer prevention, and innovations in screening, diagnosis and treatment.

## Supplementary Information

Supplementary Information.

## Data Availability

The New South Wales Cancer Registry is the data custodian for the tabulated colon and rectal cancer mortality data that support the findings of this study. Approved release of these data can be obtained through an application to the New South Wales Cancer Registry. Details are available at https://www.cancer.nsw.gov.au/. The tabulated data on colon and rectal cancer incidence, mortality and relative survival are available at https://www.aihw.gov.au/reports/cancer/cancer-data-in-australia/contents/summary.

## Abbreviations

ABS: Australian Bureau of Statistics
AIHW: Australian Institute of Health and Welfare
APC: Age-period-cohort
ICD: International Classification of Diseases
NSW: New South Wales
NSWCR: New South Wales Cancer Registry
NBCSP: Australian National Bowel Cancer Screening Program
RBDM: Registries of Births, Deaths and Marriages
