# Supplementary material for: Trends in colon and rectal cancer mortality in Australia from 1972 to 2015 and associated projections to 2040
Source: Sci Rep. 2022 Mar 7;12:3994. doi: 10.1038/s41598-022-07797-x (PMC8900106; doi:10.1038/s41598-022-07797-x)
Supplement: Supplementary file 1 — Supplementary Information. [file 41598_2022_7797_MOESM1_ESM.pdf]

Supplementary material accompanying the article:

Trends in colon and rectal cancer mortality in Australia from 1972-2015 and associated projections to 2040

Qingwei Luo,[1] Jie-Bin Lew,[1] Julia Steinberg,[1] Joachim Worthington,[1] Xue Qin Yu,[1] Michael Caruana,[1] Isabelle Soerjomataram,[2] Freddie Bray,[2] Sheena Lawrance,[3] Maria Arcorace,[3] Dianne L O'Connell,[1,4] Karen Canfell,[1] Eleonora Feletto[1]

1. The Daffodil Centre, The University of Sydney, a joint venture with Cancer Council NSW, Sydney, New South Wales, Australia.
2. Cancer Surveillance Branch, International Agency for Research on Cancer, Lyon, France
3. New South Wales Cancer Registry, Cancer Institute NSW, Sydney, New South Wales, Australia
4. School of Medicine and Public Health, University of Newcastle, Newcastle, New South Wales, Australia.

Table of Contents

Appendix 1. The collection and processing of cause of death data for the Australian Bureau of Statistics (ABS) and the New South Wales Cancer Registry (NSWCR) databases 3

Cancer mortality data in the Australian Bureau of Statistics 3

Cancer mortality data in the New South Wales Cancer Registry 3

Appendix 2. Comparison of observed age-standardised mortality rates for colon and rectal cancers using data from the Australian Bureau of Statistics (ABS) and the New South Wales Cancer Registry (NSWCR) 5

Appendix 3: Age-period-cohort (APC) model effects for colon and rectal mortality rates......... 6

Appendix 4: Observed and predicted age-standardised mortality rates for colon and rectal cancers by sex and age group in Australia, 1972 to 2040 9

Appendix 5: Comparison of the relative change in age-standardised mortality rates by world region and country published by Araghi et al., 2019 for colon and rectal cancers.................. 10

Appendix 6: Trends in incidence and mortality rates (1982-2015), and 5-year relative survival (1988-2017) for colon and rectal cancers in Australia.. 11

Appendix 7: Comparison of age-standardised incidence and mortality rates for major cancers and all cancers combined in Australia and New South Wales.. 12

References 14

Appendix 1. The collection and processing of cause of death data for the Australian Bureau of Statistics (ABS) and the New South Wales Cancer Registry (NSWCR) databases

Cancer mortality data in the Australian Bureau of Statistics

The flow chart in Figure S1.1 outlines the Australian Cause of Death Statistics System for cancer mortality data released by the Australian Bureau of Statistics (ABS), which are sourced from death registration systems administered by the various state and territory Registries of Births, Deaths and Marriages (RBDMs). It is a legal requirement of each state and territory that all deaths are registered. Information about the deceased is supplied by a relative or other person acquainted with the deceased, or by an official of the institution where the death occurred. As part of the registration process, information on the cause of death is either supplied by the medical practitioner certifying the death on a Medical Certificate of Cause of Death, or supplied as a result of a coronial investigation. The information is provided to the ABS by individual registries for coding and compilation into aggregate statistics. In addition, the ABS supplements these data with information from the National Coronial Information System (NCIS). The ABS processes, codes and validates this information, and the data quality depends on the information available from the death certificates.[1]

Cancer mortality data in the New South Wales Cancer Registry

Causes of death for cancer patients in the New South Wales Cancer Registry (NSWCR) are sourced from RBDMs and the ABS by matching registered cancer cases against death records. According to the NSW Health policy directive, if a patient presents for a consultation or treatment at any facility in NSW and has a diagnosis of cancer then the NSWCR must be notified.[2] The NSWCR employs a variety of routine data quality control measures including reconciliation of information on cancer diagnosis and cancer treatment from multiple sources, collaboration with medical experts, and examination of multiple registrations (Figure S1.1).[3] Although these measures may not be a complete /comprehensive mortality data quality assessment, they can greatly improve the quality of cause of death information.

Figure S1.1. The collection and processing of cause of death data for Cancer Data in Australia and the New South Wales Cancer Registry database

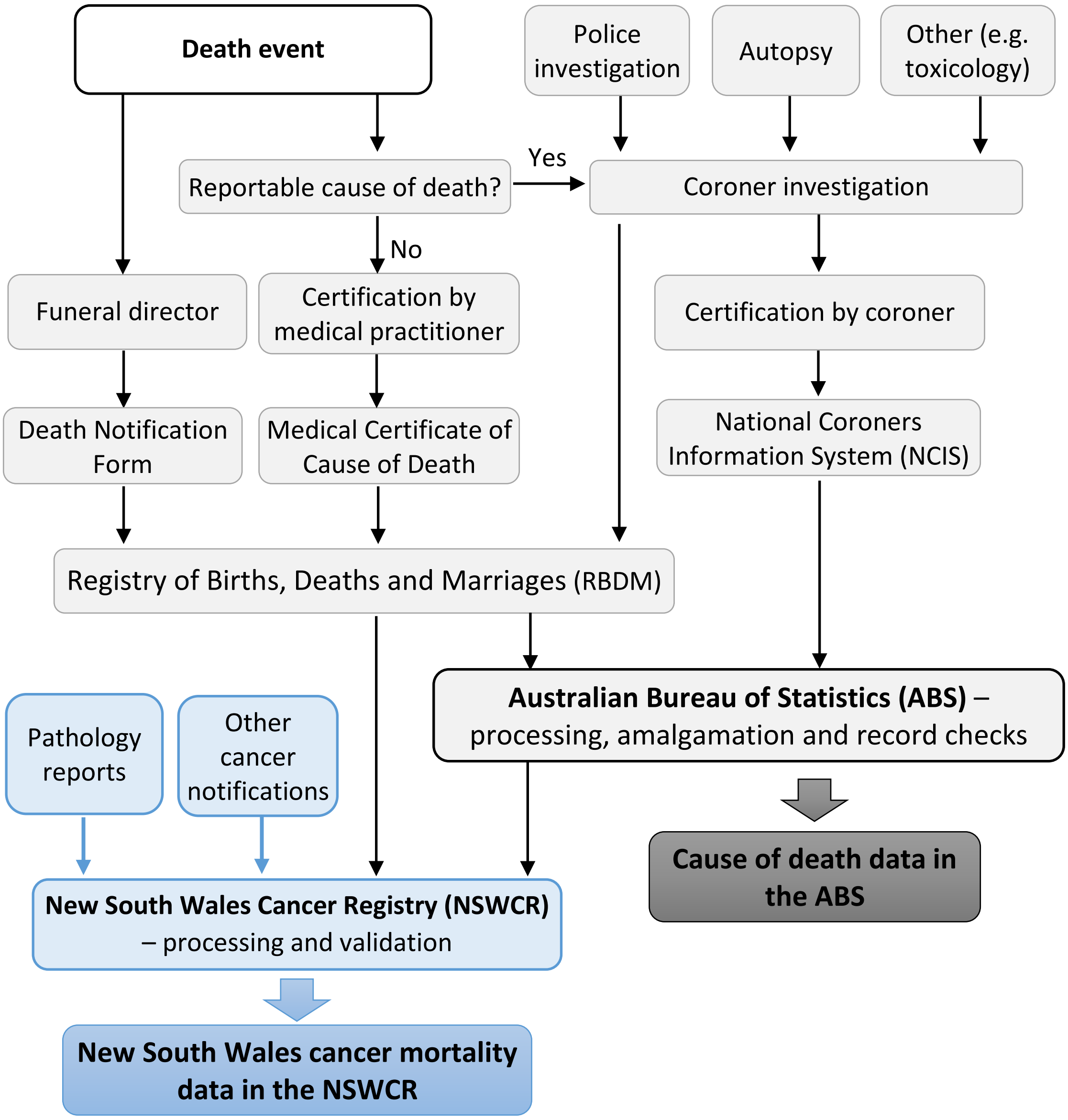

Appendix 2. Comparison of observed age-standardised mortality rates for colon and rectal cancers using data from the Australian Bureau of Statistics (ABS) and the New South Wales Cancer Registry (NSWCR)

Figure S2.1 shows the comparison of trends in age-standardised mortality rates using data from the ABS and NSWCR for colorectal cancer and by sub-site. The mortality rates for colorectal cancer using both sources are generally consistent and decreased from 1990 to 2015. The slightly higher colorectal cancer mortality rates estimated using data from the ABS are likely due to a small number of deaths coded as C26.0 not being due to colorectal cancer while these were excluded in the data from the NSWCR due to the additional validation processes.[3 4] Despite this overall agreement, mortality rates for colon and rectal cancers separately differed between the two sources. For colon cancer, mortality rates from both data sources decreased. The decline seen in the NSWCR data occurred at a slower pace after the 2000s compared to that seen in the ABS data. For rectal cancer, the mortality rate showed a steady decline from the 1990s onwards using the NSWCR data but an increasing trend using the ABS data.

Figure S2.1: Comparison of observed age-standardised mortality rates for colon and rectal cancers using data from the Australian Bureau of Statistics (ABS) and the New South Wales Cancer Registry (NSWCR)

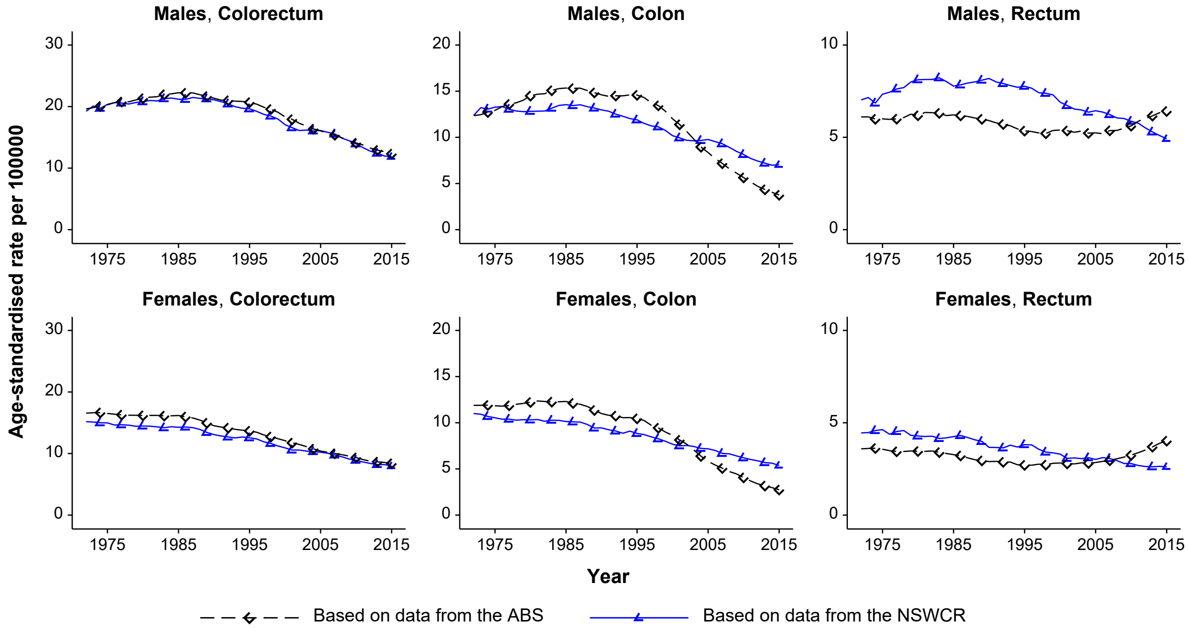

Appendix 3: Age-period-cohort (APC) model effects for colon and rectal mortality rates

The basic APC model with the log-link function can be expressed as:

$$lnD_{ij} = lnN_{ij} + \alpha_i Age_i + \beta_j Period_j + \gamma_k Cohort_k$$

where D_{ij} denotes the number of deaths for the ith age group during the jth calendar period; N_{ij} denotes the number at risk in the population for the ith age group during the jth calendar period; α_i is the coefficient of the age component for age group *i*; β_j is the non-linear coefficient of the period component for period *j*, and γ_k is the non-linear coefficient of the cohort component for birth cohort k. APC models were fitted by the apcspline command in Stata 16 with natural cubic splines for smoothing for colon and rectal cancers separately by sex and <50 years and 50 years and over.[5] The most appropriate statistical projection model with the lowest Bayesian information criterion (BIC) was selected. To project mortality rates beyond the observed period, future periods and cohorts were assumed to have the same effect as those for the most recent observed period and cohort. As these historical trends will not continue indefinitely, the default setting for the damping factor (equal to 0.92) was used, so that the drift was reduced by 8% for each year following the last observation.[5]

There is a non-identifiability problem inherent in APC models due to the linear relationship between age, period and cohort,[6] and there is no way to distinguish between the period effect and the cohort effect. The parameter estimates obtained can be sensitive to the choice of constraints placed on the period and cohort factors in each stratified model.[7] The age, period and cohort effects in the two age-stratified APC models are therefore not directly comparable between APC models. However, the observed opposing trends in cohort effects for ages <50 and 50+ resulted in different patterns in mortality rates between the two age groups for both colon and rectal cancer.

Figure S3.1. Model effects for mortality rates of colon cancer

(a) age<50 years

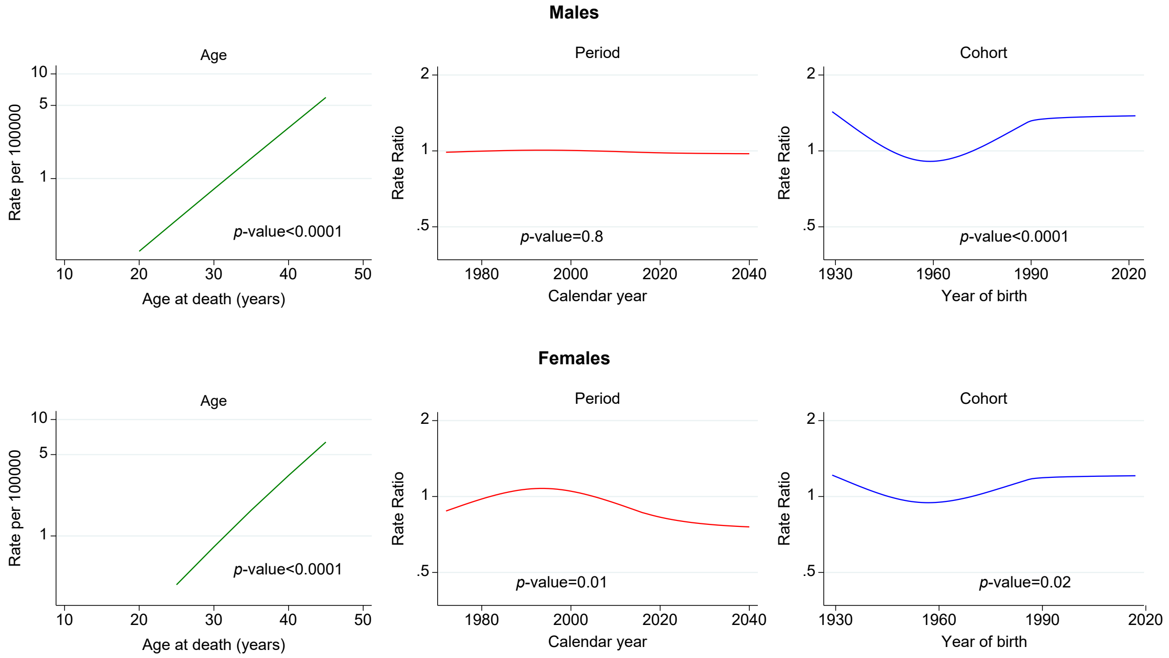

(b) age 50+ years

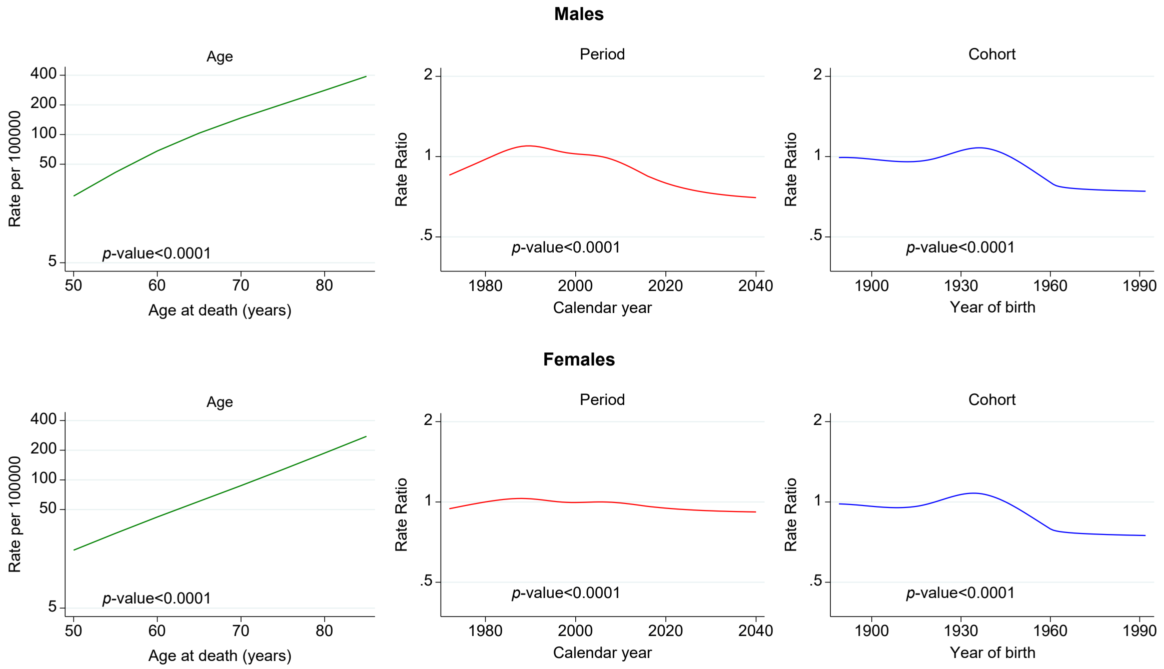

Figure S3.2. Model effects for mortality rates of rectal cancer

(a) age<50 years

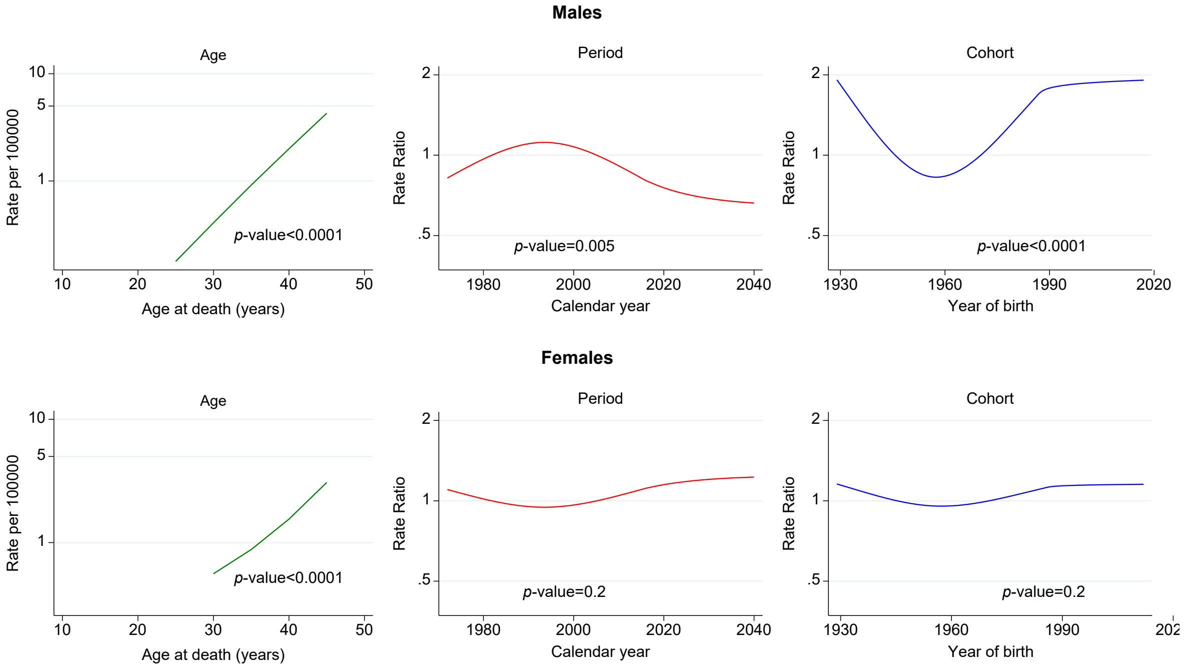

(b) age 50+ years

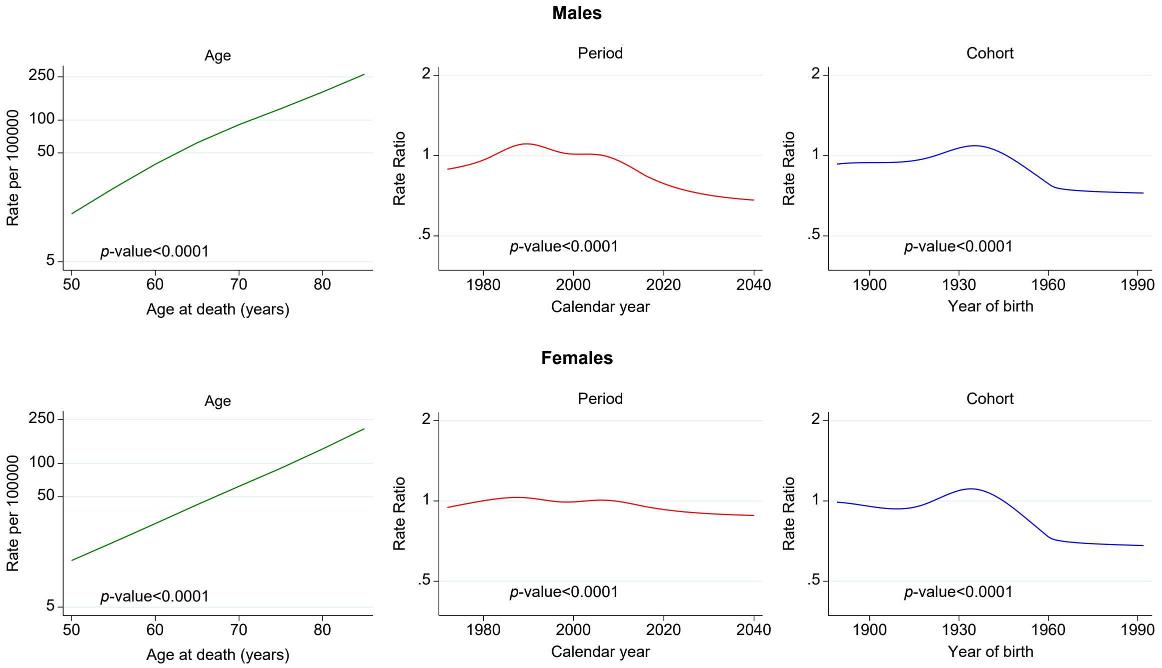

Appendix 4: Observed and predicted age-standardised mortality rates for colon and rectal cancers by sex and age group in Australia, 1972 to 2040

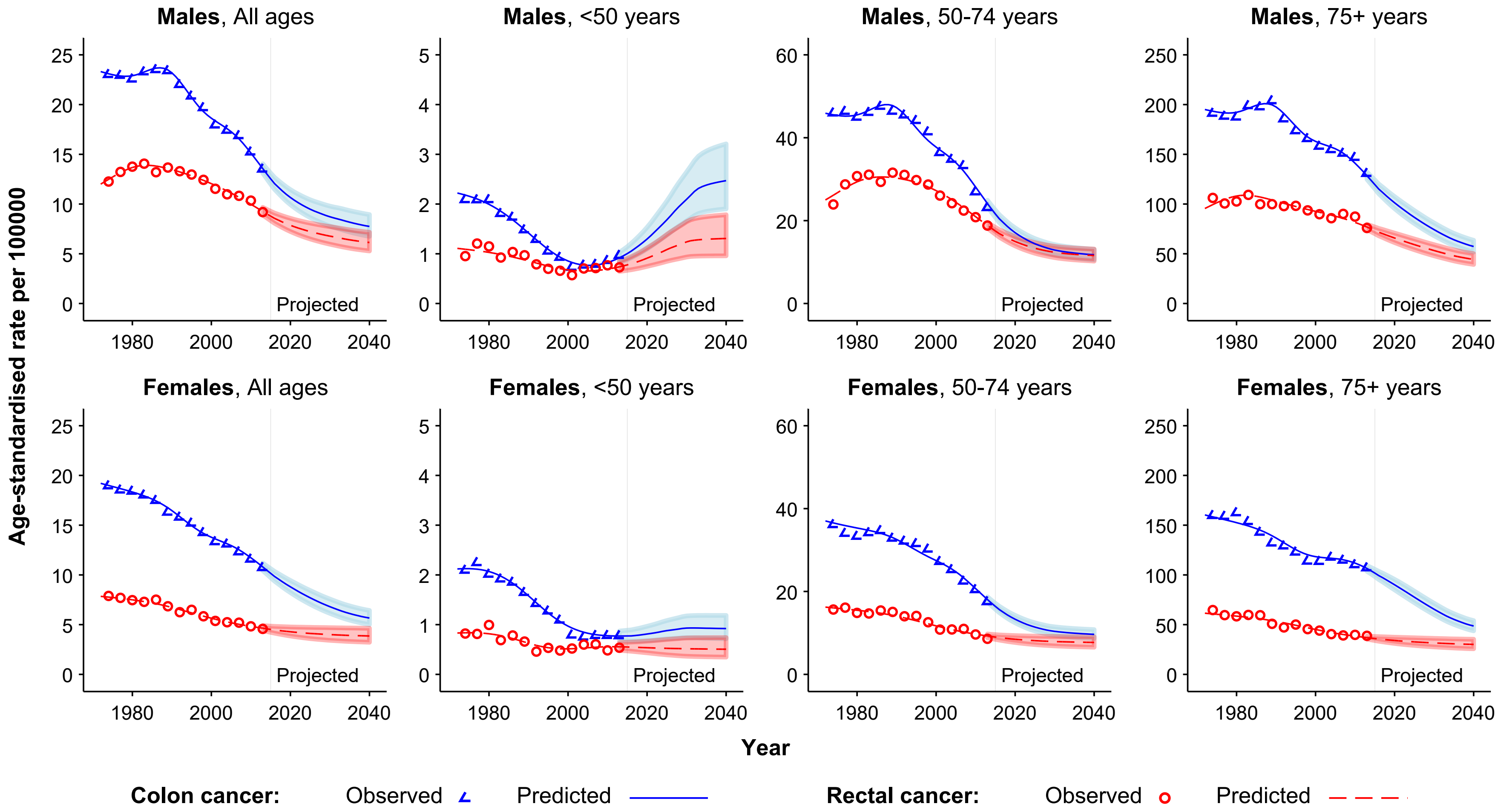

All rates are age-standardised using the 2001 Australian population.

Appendix 5: Comparison of the relative change in age-standardised mortality rates by world region and country published by Araghi et al., 2019 for colon and rectal cancers

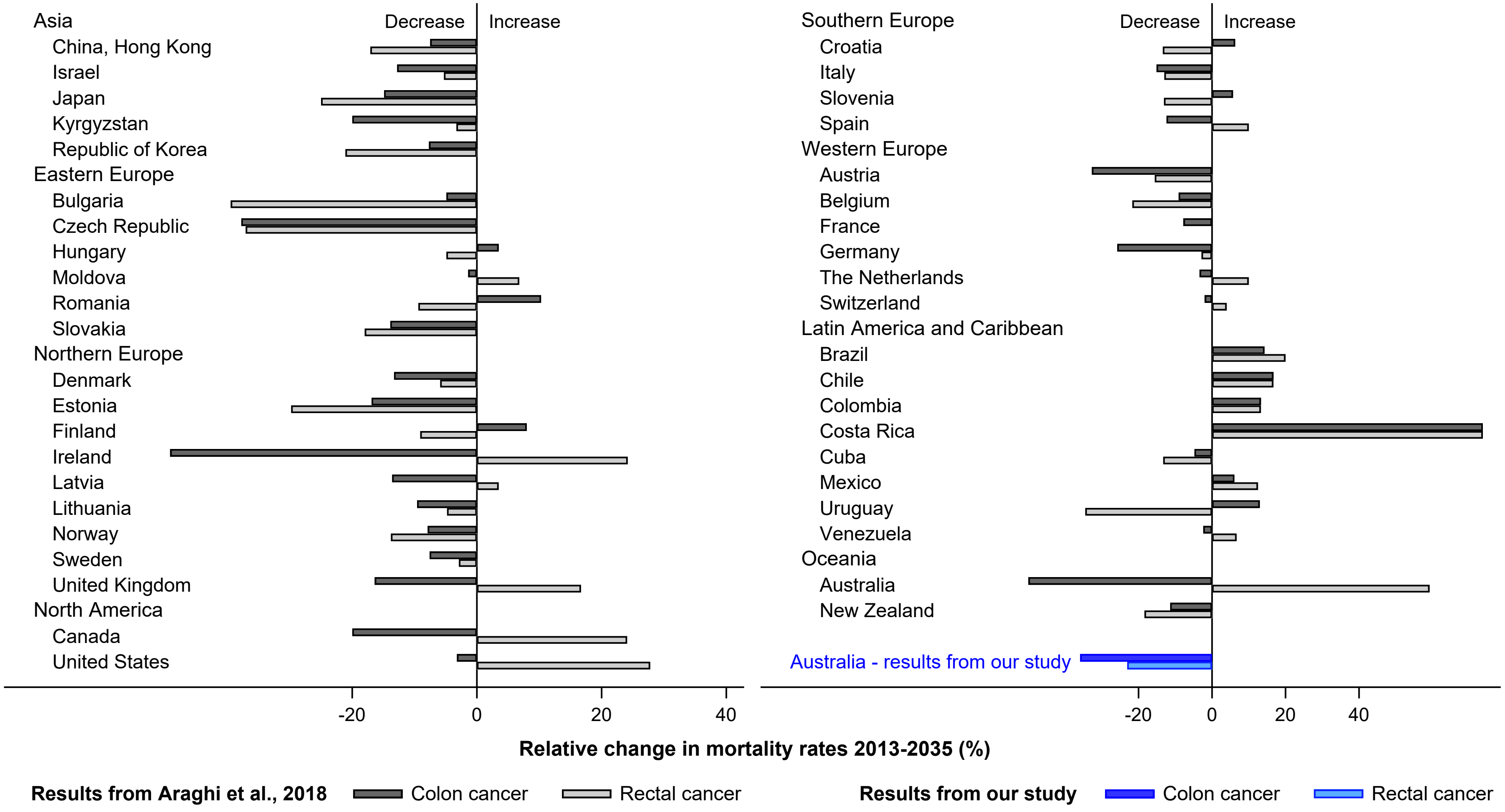

Relative change in age-standardised mortality rates from Araghi et al., 2019 were extracted from tables 1 and 2.

Reference: Araghi M, Soerjomataram I, Jenkins M, et al. Global trends in colorectal cancer mortality: projections to the year 2035. Int J Cancer 2019.

Appendix 6: Trends in incidence and mortality rates (1982-2015), and 5-year relative survival (1988-2017) for colon and rectal cancers in Australia

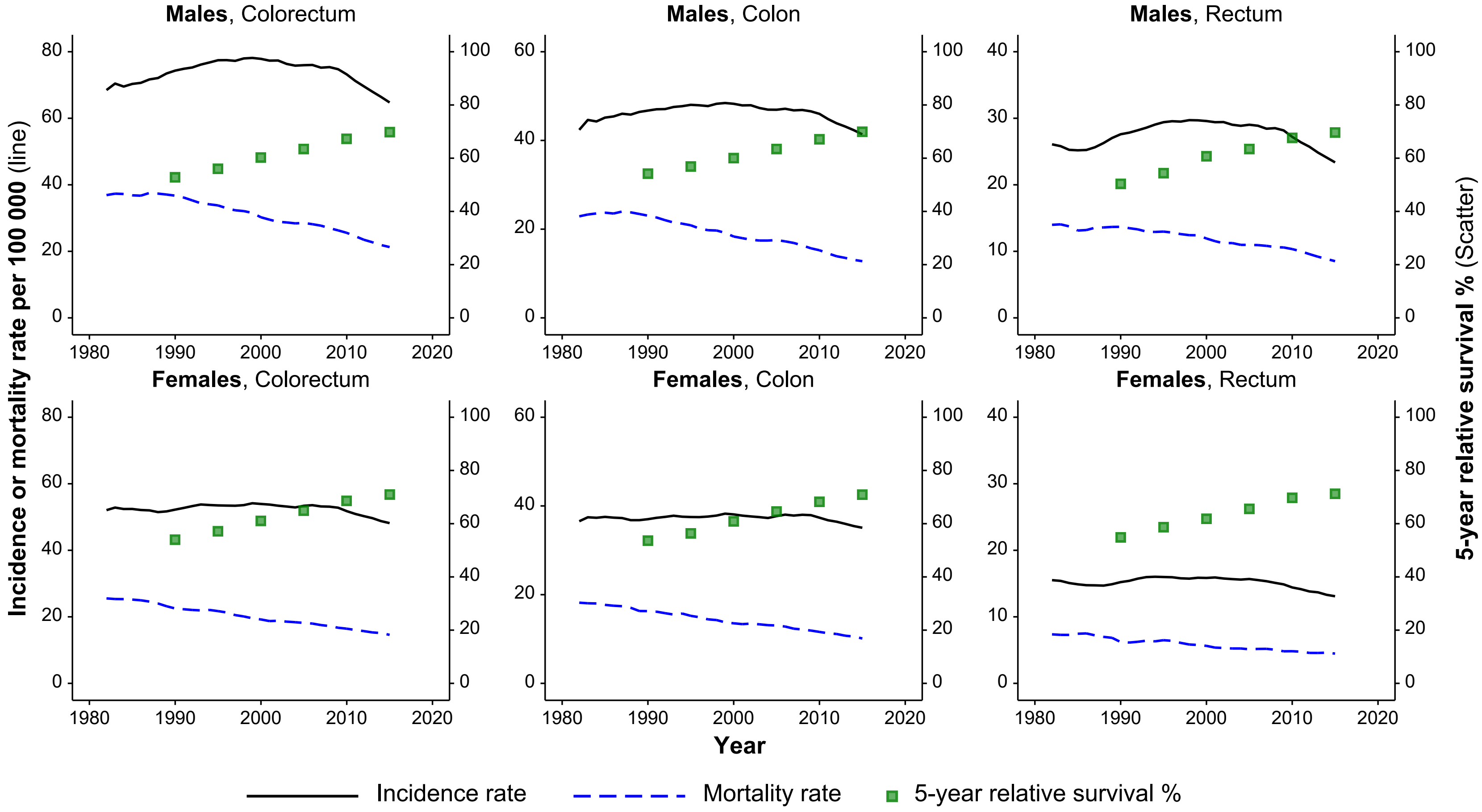

Incidence rates and 5-year relative survival for Australia were sourced from the Australian Institute of Health and Welfare. Mortality rates for colon and rectal cancer were based on the New South Wales Cancer Registry data. All rates are age-standardised using the 2001 Australian population.

Appendix 7: Comparison of age-standardised incidence and mortality rates for major cancers and all cancers combined in Australia and New South Wales

A. Incidence rates

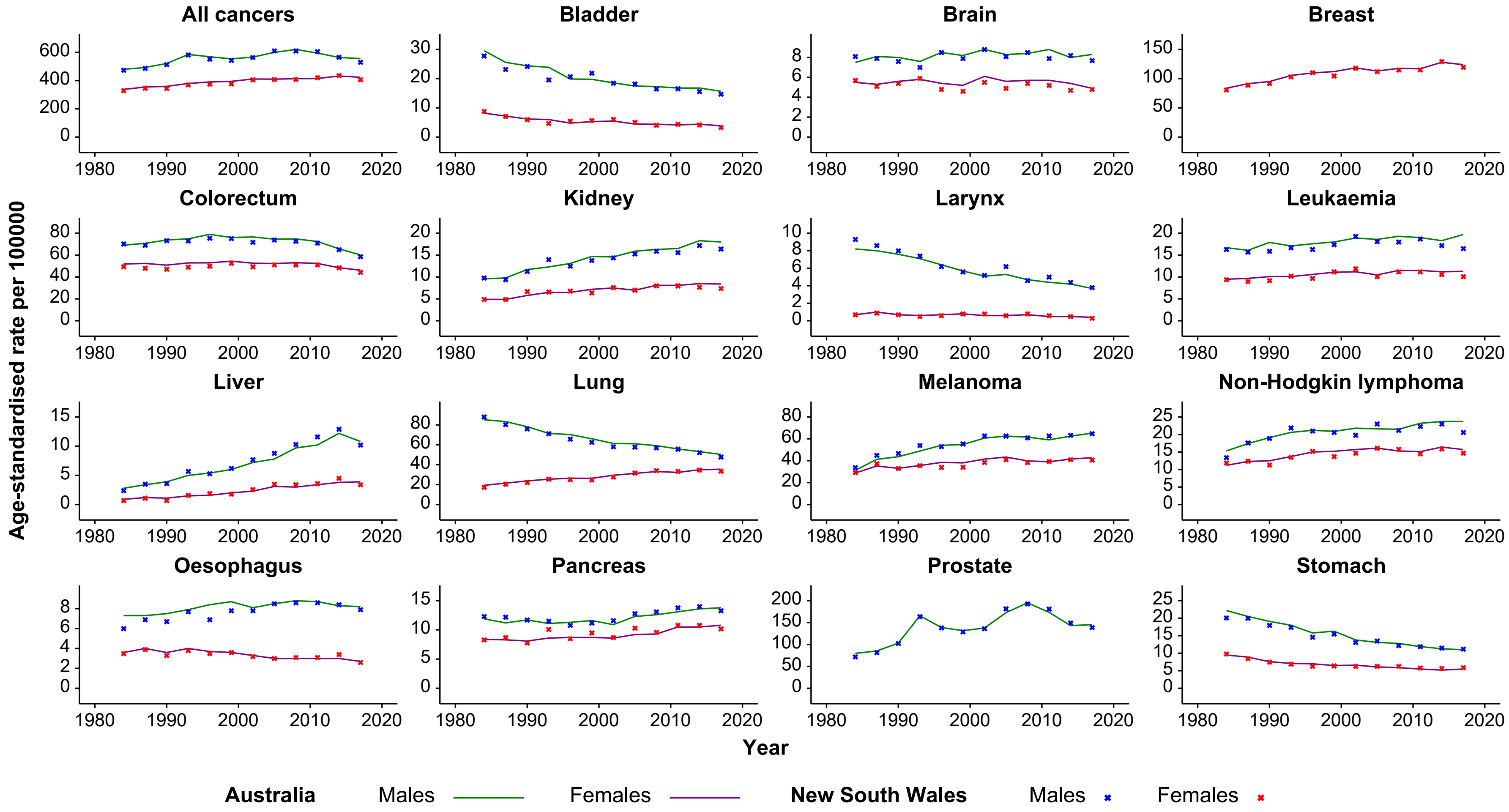

B. Mortality rates

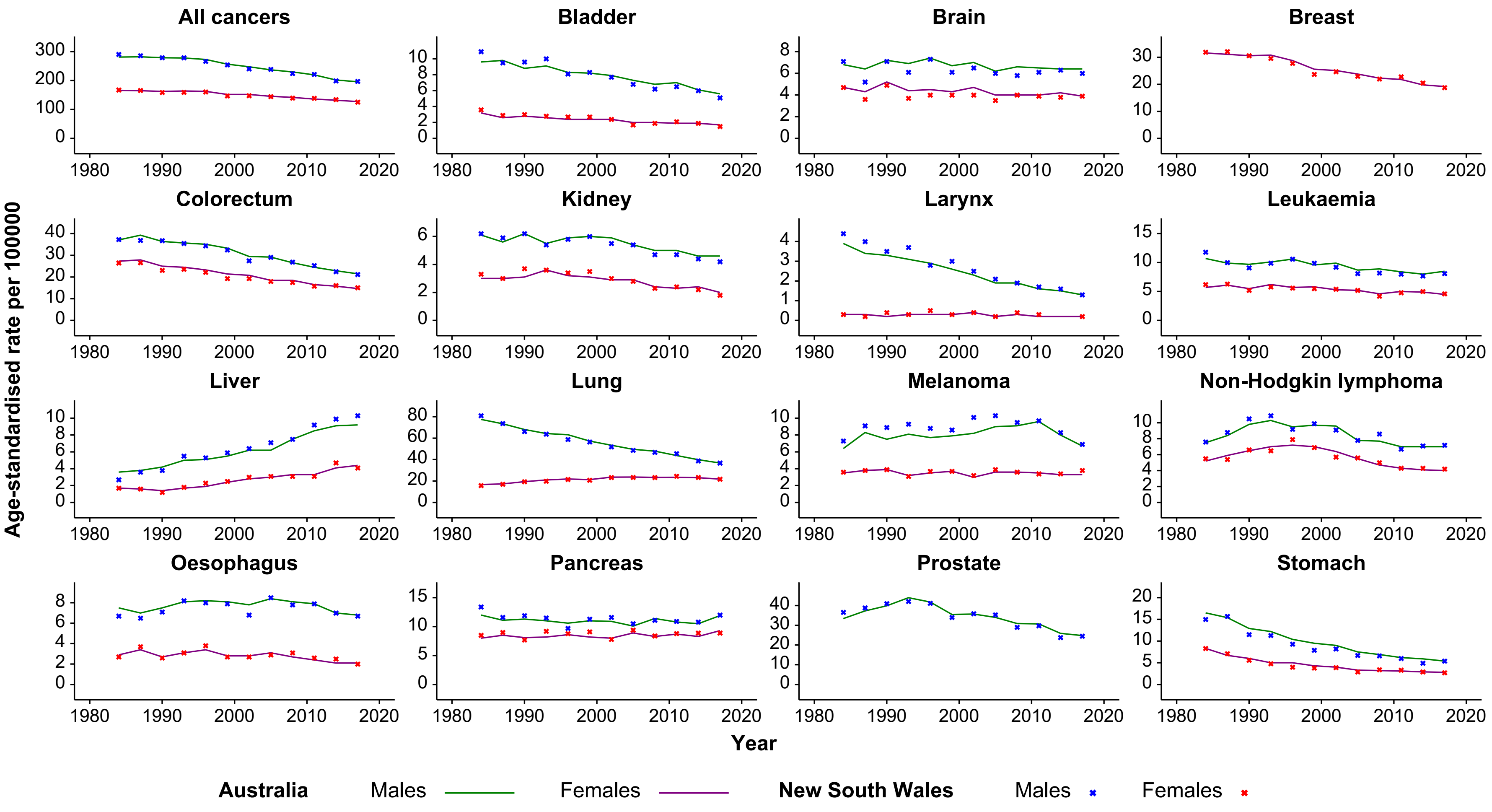

Data sourced from the Australian Institute of Health and Welfare 2021. (Reference: Australian Institute of Health and Welfare, 2021.[8]) All rates are age-standardised using the 2001 Australian population.

References

1. Australian Bureau of Statistics. Causes of Death, Australia methodology. ABS, Canberra., 2019.
2. Department of Health NSW. Cancer registry - notifying cancer cases to the NSW Central Cancer Registry In: Research Ea, ed., 2009.
3. NSW Cancer Registry. Data quality, incidence and mortality data 2021 [Available from: https://www.cancer.nsw.gov.au/research-and-data/cancer-data-and-statistics/request-unlinked-unit-record-data-for-research/nsw-cancer-registry accessed 12 July 2021.
4. Australian Bureau of Statistics. Complexities in the measurement of bowel cancer in Australia, Cause of Death, Australia, cat. no. 3303.0. Canberra, Australia, 2016.
5. Sasieni P. Age-period-cohort models in Stata. *The Stata Journal* 2012;12(1):15.
6. Clayton D, Schifflers E. Models for temporal variation in cancer rates. I: Age-period and age-cohort models. *Stat Med* 1987;6(4):449-67. [published Online First: 1987/06/01]
7. Kupper LL, Janis JM, Karmous A, et al. Statistical age-period-cohort analysis: a review and critique. *J Chronic Dis* 1985;38(10):811-30.
8. Australian Institute of Health and Welfare (AIHW). Cancer data in Australia. Cat. no: CAN 122. 8 June 2021 ed, 2021.
